# Sex-Specific Adherence to the Mediterranean Diet in Obese Individuals

**DOI:** 10.3390/nu16183076

**Published:** 2024-09-12

**Authors:** Laura Di Renzo, Paola Gualtieri, Giulia Frank, Rossella Cianci, Glauco Raffaelli, Daniele Peluso, Giulia Bigioni, Antonino De Lorenzo

**Affiliations:** 1 Section of Clinical Nutrition and Nutrigenomic, Department of Biomedicine and Prevention, University of Rome Tor Vergata, Via Montpellier 1, 00133 Rome, Italy; 2 PhD School of Applied Medical-Surgical Sciences, University of Rome Tor Vergata, Via Montpellier 1, 00133 Rome, Italy; 3 School of Specialization in Food Sciences, University of Rome Tor Vergata, Via Montpellier 1, 00133 Rome, Italy; 4 Department of Translational Medicine and Surgery, Catholic University of the Sacred Heart, 00168 Rome, Italy; 5 Fondazione Policlinico Universitario A. Gemelli, Istituto di Ricovero e Cura a Carattere Scientifico (IRCCS), 00168 Rome, Italy; 6 Department of Biology, University of Rome Tor Vergata, Via Montpellier 1, 00133 Rome, Italy

**Keywords:** mediterranean diet, obesity, MEDAS, sex difference

## Abstract

Adherence to the Mediterranean diet (MedDiet) has long been associated with several health benefits, including a reduced risk of heart disease, diabetes, and obesity. The MedDiet is characterized by a high consumption of foods such as fruits, vegetables, whole grains, fish, and olive oil, along with a moderate intake of red meat and red wine with meals. Some studies report significant differences between men and women in susceptibility to obesity, with women at a higher prevalence of obesity than men. One unexplored aspect, however, concerns the sex difference in MedDiet adherence, which could be influenced by various factors, such as health perceptions, food preferences, and cultural influences. The aim of this study is to assess the effectiveness and impact of MedDiet adherence in men and women, with a focus on its influence on health and well-being, as well as its ability to promote sex equity in healthcare outcomes. Moreover, we aim to measure the overall health improvements in men and women participating in a MedDiet program, including changes in body composition and overall quality of life. This study highlights that the MedDiet is associated with more significant body weight loss in women, although their increase in MedDiet adherence was lower than in men. Trial registration: NCT01890070. Registered 24 June 2013.

## 1. Introduction

The consumption of a healthy diet is one of the most important modern challenges, mostly due to its involvement in sustainable food systems [1]. The literature data have well established the benefits of the Mediterranean diet (MedDiet) on health. The MedDiet is characterized by a high intake of fish, plant-based foods, nuts, and seeds, a limited intake of red meat, saturated fat, and processed foods, and the principal source of fat represented by monounsaturated ones, such as olive oil [2]. The MedDiet can apport beneficial effects on well-being, and adherence to MedDiet plays a pivotal role in achieving better results in terms of health and quality of life. Sex represents a key factor in food choices, due to differences between sexes in eating behaviors and health motivation [3]. Moreover, sex differences in dietary adherence are influenced by physiological factors, such as hormone regulation and non-homeostatic factors like food cravings, with women often driven by emotional triggers and men by positive reinforcement. Men tend to favor meat and dine out more often, while women prefer healthier foods and eat more frequently throughout the day [4]. Del Campo et al. [5] observed that food neophobia, particularly towards vegetables, meat, fish, pulses, game meat, and fruits, was more prevalent among women who were overweight or obese. Economic status and smoking were inversely associated with food neophobia, especially towards healthy foods, with these associations being more pronounced in men than in women.

It has been reported that women have higher MedDiet adherence [6] and lower dropout rates than men [7], but the data are still controversial [8,9]. Indeed, Leblanc et al. [10] have reported that the MedDiet leads to more beneficial changes in long-term dietary intakes with a better anthropometric and metabolic profile in men than in women. These changes were related to the fact that baseline dietary habits in women are in line with the nutrient intake of the MedDiet. Moreover, some MedDiet nutrients can modulate the brain’s serotonin pathway [11]. The hormonal changes that occur during adolescence influence sex differences in the development of fat mass (TBFat) and muscles. It has been shown that adolescents with poorer adherence to the MedDiet present higher systemic inflammatory markers linked to the risk of developing metabolic syndrome, obesity, and insulin resistance [12]. Moreover, differences between men and women in healthy habits have been shown in the case of lower adherence to the MedDiet, but no differences between the sexes have been shown if adherence to the MedDiet was optimal [13]. Furthermore, the MedDiet is associated with a significant difference between women and men in cardiovascular diseases. In particular, it was observed that after 4 weeks of MedDiet adherence, both sexes showed significant improvements in plasma lipid profiles, but only men demonstrated notable enhancements in insulin homeostasis [14]. Bédard et al. [15] found that while both men and women experienced similar reductions in systemic inflammation from following the MedDiet, this effect was only influenced by overall inflammatory status in men. Additionally, the MedDiet has shown a reduced risk of overall cause mortality in cardiovascular events in women [16].

It is also well known that the MedDiet plays a crucial role in fighting obesity, which, as per the World Health Organization, is characterized by abnormal or excessive fat accumulation, posing an elevated health risk [17]. Obesity is a chronic disease and a significant public health issue, impacting both mental and physical well-being. The prevalence of obesity is on the rise globally, cutting across age, race, and socioeconomic status [18], and a higher prevalence in women than in men is reported [19]. Several studies have been conducted in classifying obesity phenotypes, in relation to the percentage of TBFat (PBF) and its effect on metabolic health [20]. However, to date, whether there are sex differences in the effects of the MedDiet in counteracting obesity has not been investigated.

Thus, the first aim of this study was to assess whether there are sex differences in MedDiet adherence, and also in relation to age. The second aim was to evaluate if MedDiet adherence differently affects body weight (BW), body mass index (BMI), and body composition, according to sex.

## 2. Materials and Methods

### 2.1. Study Design

We conducted two different protocol studies at the Section of Clinical Nutrition and Nutrigenomics, Department of Biomedicine and Prevention at the University of Rome Tor Vergata. 

The first protocol provided the analysis of data previously collected by a web-based survey on a wide sample of subjects, from the “Design model of the network of food safety, nutritional quality and nutrigenomics systems of the Mediterranean Diet for health defense in Italy: application of the Nutrient Analysis of Critical Control Point process (MOOD)” project. The following information was obtained from previous web-based surveys [21]: (1) personal data (sex at birth; age; lifestyle); (2) anthropometric information (reported BW and height); and (3) dietary habit information: questionnaires on food frequency (FFQ) [22] and on adherence to the MedDiet, using the validated 14-item Mediterranean diet adherence screener (MEDAS) [23]. FFQ investigates the weekly frequency consumption of 36 different foods commonly consumed in Italy, and their portion sizes [22]. MedDiet adherence was classified into three groups based on the following MEDAS values: low adherence (score 0–5), medium (score 6–9), and high (score ≥  10). Compliance rates for each food were calculated.

The second study was an interventional pilot study involving 94 healthy subjects who provided informed consent for a clinical trial. These subjects were consecutively recruited as part of a routine medical examination program from September 2023. Informed consent was obtained according to the 1975 Declaration of Helsinki, revised in 2013, and the study was approved by the Ethics Committee of the Calabria Region Central Area Section (protocol no. 97, 20 April 2023).

All participants, after a baseline evaluation, received 8 weeks (8 wks) of personalized MedDiet intervention. The personalized MedDiet was tailored to personal energy requirements, calculated using the De Lorenzo formula based on lean body mass (LBM) [24]. FFQ [22] and MEDAS [23] questionnaires were employed to determine the weekly frequency of various food items and to test MedDiet adherence at baseline and after the intervention. 

To be eligible, individuals needed to fulfill the following specified inclusion criteria: ranging in age from 18 to 65 years, with a BMI ≥18.5 kg/m^2^ and <40 kg/m^2^, and self-sufficient subjects, able to feed themselves and read and understand the questionnaire in its entirety. The exclusion criteria encompassed conditions such as pregnancy, active smoking, blood pressure ≥140/90 mmHg, BMI < 18.5 kg/m^2^ and ≥40 kg/m^2^, acute illnesses, autoimmune and intestinal disorders, HIV/AIDS, neoplasms, infectious diseases, alcohol and drug abuse, and adherence to a vegetarian or vegan diet. It is noteworthy that all participants were required to be in a state of good health and without chronic illness. Participants were instructed to maintain regular physical activity and lifestyle throughout the study, reporting any instances of illness or abnormalities. Biweekly telephone interviews were conducted to obtain a nutritional recall of the participants’ dietary intake over the previous 24 h, assessing MedDiet adherence and addressing complications or adverse effects. After each treatment period, a trained medical doctor evaluated potential adverse effects using a checklist of symptoms. All participants completed the study successfully, with no alterations to trial outcomes after commencement.

### 2.2. Mediterranean Diet Intervention

The daily macronutrient composition of the MedDiet was delineated as follows: 40–45% of the total daily caloric intake (kcal/day) from carbohydrates, 25–30% from proteins (with more than 50% sourced from vegetables), 25–30% from lipids (with polyunsaturated fatty acids (PUFAs) at 6–10%, a *n*-6/*n*-3 PUFA ratio of 3:1, monounsaturated fatty acids (MUFAs) at 15%, trans-fatty acids at <1%, and saturated fat at <10%), and a fiber intake of 25 g. The distribution of calorie intake throughout the day was as follows: 15% for breakfast, 10% for the morning snack, 35% for lunch, 10% for the afternoon snack, and 30% for dinner.

The Mediterranean Adequacy Index (MAI) was computed to evaluate an individual’s adherence to the MedDiet based on specific dietary components typical of this pattern. The MAI was determined and classified as described previously [25].

### 2.3. Assessment of Body Composition

Anthropometric and body composition parameters were examined at baseline and after 8 wks of MedDiet intervention. BW, height, and waist circumference were measured as described previously [26]. The body mass index (BMI) was computed as BW (kg)/height (m^2^) and categorized, according to Di Renzo et al. [20], as follows: normal weight (NW), normal weight obese (NWO), metabolically obese normal weight (MONW), metabolically healthy obese (MHO), and metabolically unhealthy obese (MUO).

A BIA phase-sensitive system (BIA 101S, Akern/RJL Systems, Florence, Italy) was utilized to assess resistance, reactance, impedance, the phase angle (PhA), and body cell mass (BCM) at a 50 kHz frequency, as described previously [27]. 

An X-ray densitometer (DXA) (Primus, X-ray densitometer; software version 1.2.2, Osteosys Co., Ltd., Guro-gu, Seoul, Republic of Korea) was employed at baseline, as described previously [27], for body composition analysis, encompassing soft tissues, TBFat, and LBM.

### 2.4. Statistical Analysis

Data were collected using Microsoft Office Excel^®^ (2020, Microsoft, Redmond, WA, USA). Statistical analyses were conducted utilizing IBM SPSS version 21.0 for Windows (IBM Corp. in Armonk, NY, USA). After performing a Shapiro–Wilk test, we applied either an unpaired *t*-test or a nonparametric Wilcoxon or Kruskal–Wallis test to evaluate changes at baseline and after 8 wks of MedDiet treatment and any variations between the two sexes.

The differences in parameter levels (delta) were computed as follows: Δ% = ((Z − W)/W) × 100, where Δ% represents the percentage variation of the parameter, calculated as the ratio of absolute variation to the baseline value (W); Z is the value of the parameter after treatment, and W is the value of the parameter at baseline.

Pearson’s Chi-square or Spearman’s rank correlation tests were executed to explore associations or correlations between categorical variables and to understand possible influences of confounding factors, such as age and BMI. The results are presented as the mean standard error of the mean (S.E.M.). The null hypothesis was rejected at the 0.05 probability level in all conducted statistical tests.

## 3. Results

The first protocol concluded with the collection and analysis of data for a total of 4403 participants who completed the questionnaire. The female respondents represented 75.24%, while the male respondents represented 24.74%. The general and anthropometric characteristics of the population are shown in Table 1.

**Figure 1:**
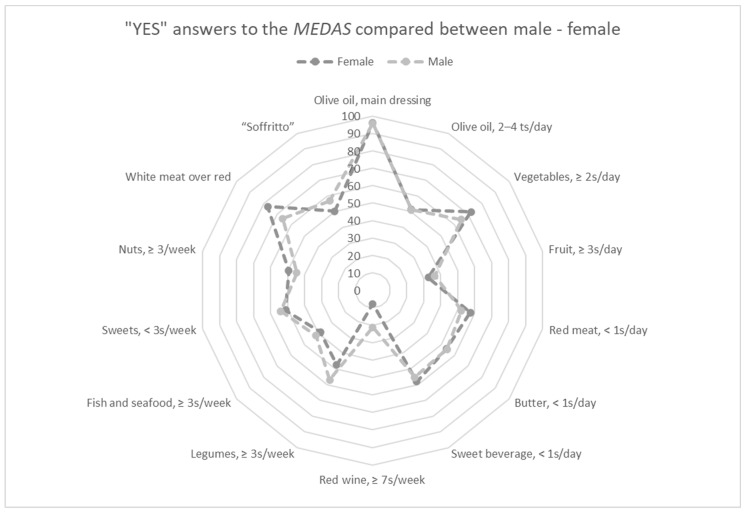
Answers and scoring of the MEDAS. The “YES” answer to the MEDAS items is compared between the two sexes. The radar chart shows for each MEDAS score item the affirmative responses of males compared to females with separate axes starting from the center of the draft (0% “YES” responses) and at the end of the last circle (100% “YES” responses). Values are in percentages.

Among the MedDiet adherence groups, no significant differences were observed between the two sexes. However, after performing a Pearson’s Chi-square test, significant associations between several MEDAS items and the two sexes were reported. Particularly, significant associations were shown for vegetables (*p* < 0.0001), fruits (*p* < 0.02), red meat (*p* < 0.002), red wine (*p* < 0.0001), legumes (*p* < 0.0001), fish and seafood (*p* < 0.04), nuts (*p* < 0.008), white meat over red (*p* < 0.0001), and “soffrito” (*p* < 0.0001). The results of the answers and the scoring of the MEDAS are reported in Table 2 and Figure 1.

A Pearson’s Chi-square test was conducted to verify if there was any association between men and women according to MedDiet adherence and age groups. No statistically significant associations were observed in MedDiet adherence groups (*p* < 0.21) and <18 (*p* < 0.74), 18–30 (*p* < 0.55), 31–64 (*p* < 0.70), and >64 (*p* < 0.25) groups. 

A non-parametric Kruskal–Wallis test was performed to verify if there were any differences in the MedDiet adherence groups on anthropometric parameters. A significant difference was observed in BW (*p* < 0.0001) and BMI (*p* < 0.0001). Moreover, a significant dependence between MedDiet adherence and BW for women was shown (*p* < 0.0001), but not for men (*p* < 0.45). 

To verify the effect of MedDiet adherence, the smallest sample was analyzed at baseline and after 8 wks of MedDiet treatment. Body composition characteristics and obesity classification at baseline are shown in Table 3.

Particularly, for the whole sample who followed the MedDiet treatment, a 19.1% increase in MEDAS score was observed after 8 weeks, with a greater median increase in men (+30%) than women (+11.1%). Moreover, an increase in high adherence (+31.9%) and a decrease in low (−12.76%) and medium (−19.15%) were shown. MedDiet adherence groups before and after MedDiet treatment are reported in Table 4.

A non-parametric Wilcoxon test for paired samples was performed to verify if there were any significant differences between before and after MedDiet treatment. Significant differences were observed in BW (*p* < 0.0001), BMI (*p* < 0.0001), PhA (*p* < 0.01), PBF (*p* < 0.0001), MEDAS score (*p* < 0.0001), and MedDiet adherence groups (*p* < 0.0001). No significant differences were observed in BCM% (*p* < 0.44). 

After performing a Spearman’s rank correlation rho analysis between Δ% variables, no significant correlations were observed between MedDiet adherence and BW (*p* < 0.005, ρ = −0.28), BMI (*p* < 0.006, ρ = −0.27), PhA (*p* < 0.25, ρ = 0.11), PBF (*p* < 0.08, ρ = −0.17), and BCM% (*p* < 0.01, ρ = 0.24). 

A correlation matrix was assessed to analyze trends between Δ% variables. The correlation matrix of the whole sample is reported in Table 5.

The correlation matrix for the female sample is shown in Table 6.

The correlation matrix for the male sample is shown in Table 7.

## 4. Discussion

It is well established that sex plays a key role in daily life, influencing factors such as diet, and physical activity through different perceptions of illness and well-being [28]. In addition, chronobiology and sex differences also play a critical role in regulating appetite and eating behavior [29]. On the other hand, gender-related behaviors, such as lifestyle and diet, may induce epigenetic changes that can modulate the onset of diseases [30]. It has been shown that women pay better attention to the type of food intake which is related to better diet quality [31]. Indeed, in this observational study, of 4403 participants who completed the questionnaire, 75.24% were represented by women.

Particularly, concerning the consumption of individual MEDAS items, significant variations were observed between the sexes, with women’s differences of +7% for vegetables, +4.67% for nuts, −5.21% for red meat, and +11.04% for preferring white meat over red, compared with men. However, men reported differences of +3.7% for fruit, +9.43% for legumes, +3.31% for fish and seafood, and +6.57% for “soffrito” with respect to women. These results are in agreement with Engler et al. [32] who noted that women significantly consume less fruit and wine, and more vegetables compared to men. However, Predieri et al. [33] observed that women consume more vegetables, but also more fruits and legumes, while men consume significantly more red meat and wine. Overall, these data seem to confirm that men consume more animal proteins than women [34]. 

Moreover, it is well established that the MedDiet is characterized by a low intake of alcoholic beverages [35]. In this study, men reported a difference of +13.43% for red wine consumption compared with women, according to Melguizo-Ibáñez et al. [36] who have previously shown that men have higher social use of alcoholic drinks. 

It is well known that the MedDiet is linked to a notable enhancement in health status and an increase in well-being [27]. The MedDiet demonstrated positive effects on metabolic abnormalities, resulting in reduced occurrences of metabolic syndrome and type 2 diabetes [37]. The MedDiet has the potential to reduce the morbidity associated with major chronic diseases, reducing inflammation and enhancing endothelial function and respiratory fitness. These improvements manifest as favorable alterations in atherogenic dyslipidemia, hypertension, insulin resistance, and especially, central obesity [38]. Notably, a significant difference was observed in the MedDiet adherence group concerning BW and BMI, showing that as MedDiet adherence increases, BW and BMI decrease, and vice versa. These results are in line with those observed in the interventional pilot study, in which significant differences were observed after 8 wks of MedDiet treatment for BW (*p* < 0.0001), BMI (*p* < 0.0001), and PBF (*p* < 0.0001) in both sexes. Several studies [39] have shown that increased adherence to the MedDiet promotes a significant decrease in several anthropometric and body composition parameters. Particularly, Di Daniele et al. [39] showed that a 6-month MedDiet treatment resulted in decreased BMI, BW, and TBFat, especially in the abdominal region that functions as a metabolically active organ, contributing to abnormalities associated with an elevated risk of cardiovascular disease, diabetes, and overall mortality. Concerning obesity phenotypes, in our interventional pilot study, we reported that at baseline, the NWO phenotype is more represented in women (21.74%) than in men (1.41%). These data are in line with Di Renzo et al. [40], who showed the prevalence of this phenotype in the female sex. In contrast, for the first time, we noted that the MHO phenotype is more represented in men (33.8%) than women (17.39%), while several studies reported a higher prevalence of MHO in women than men [41]. More investigations are needed on body composition in relation to sex and on a longer duration of MedDiet treatment to better understand how MedDiet adherence can impact obesity phenotypes. Indeed, MedDiet adherence is associated with a regression of visceral adiposity, and can promote the maintenance of the healthy metabolic phenotype and reversion from the unhealthy phenotype, especially in postmenopausal women [42].

MedDiet adherence in women is known to be associated with better metabolic health, as shown by Di Renzo et al. [27], enhancing the lipid metabolism profile, especially for the high-density lipoprotein. For the first time, our observational study reported a significant association between MedDiet adherence and BW only in women. These results were also confirmed in the interventional pilot study, in which negative trends were noted in BW and MEDAS score (ρ = −0.43), and BMI and MEDAS score (ρ = −0.44), and a positive trend was reported in the MEDAS score with MedDiet adherence groups (ρ = 0.47) in women. 

From the interventional pilot study, a significant mean increase of 19.1% in the MEDAS scores (*p* < 0.0001), after 8 wks of MedDiet treatment was reported, with a significant difference in MedDiet adherence groups (*p* < 0.0001) related to an increase in high adherence (+31.9%) and a decrease in low (−12.76%) and medium (−19.15%) adherence. Notably, men reported a greater median increase in MEDAS scores (+30%), starting from 7 and improving it at 10, than women (+11.1%) who started from 8 and improved it at 9. This result is in line with Leblanc et al. [10], who showed how these changes were related to the fact that baseline dietary habits in women are related to the nutrient intake of the MedDiet. Thus, dietary modifications after 8 wks of nutritional intervention were not as relevant in women as in men.

Barrea et al. [43] reported an association between MedDiet adherence and PhA, independent of sex, thus showing that in both sexes, greater MedDiet adherence results in larger PhA as an expression of cell membrane integrity. Accordingly, our interventional pilot study reported a significant difference after 8 wks of MedDiet treatment for PhA (*p* < 0.01) in both sexes. Notably, a positive trend between PhA and BCM was shown in men. Indeed, it is well established that PhA is related to cellular health and, thus to BCM% values [44].

To our knowledge, this study is the first to comprehensively investigate sex-specific differences in the effects of the MedDiet on an Italian population, providing valuable insights into dietary behaviors and metabolic outcomes. This study employed both observational and interventional approaches, enhancing the robustness of the findings. The use of validated dietary assessment tools and an 8-week nutritional intervention allowed for a detailed examination of MedDiet adherence and its impact on body composition. 

However, several limitations must be acknowledged. Firstly, the first protocol relies on a self-reported questionnaire, potentially introducing data misreporting. However, our web survey aligns with commonly used methodologies. Additionally, factors beyond diet, such as lifestyle variations, in the interventional study may contribute to observed differences. Lastly, the clinical trial was conducted at a single center and only on an Italian sample, potentially limiting the generalizability of our findings to broader populations.

Future research should include longer-term follow-ups to assess the sustained impact of the MedDiet on health outcomes. Additionally, expanding the study to multiple centers and more diverse populations could improve the generalizability of the findings. Further exploration of the relationship between MedDiet adherence and specific obesity phenotypes, particularly in relation to sex differences, is also warranted. Studies that integrate lifestyle factors, including physical activity and sleep patterns alongside dietary interventions, would provide a more holistic understanding of the role of the MedDiet in promoting health and well-being.

## 5. Conclusions

This study highlighted that the MedDiet is associated with more significant BW loss in women, although their increase in MedDiet adherence was lower than in men. 

Our discoveries could provide valuable insights for future research aiming to customize diets to individual needs, fostering improved health homeostasis and well-being.

## Figures and Tables

**Table 1:** Participants’ general characteristics and anthropometrics.

Variables	Women	Men	Whole Sample
N sample	3313 (75.24)	1090 (24.74)	4403
Height (cm)	164.5 ± 6.42	176.4 ± 7.17	167.5 ± 8.4
Weight (kg)	64.31 ± 13.15	79.58 ± 13.46	68.1 ± 14.78
BMI (kg/m^2^)	23.73 ± 4.51	25.52 ± 3.82	24.17 ± 4.42
Age (years)	39.14 ± 13.47	40.61 ± 16.74	39.5 ± 14.4
<18	93 (48.50)	97 (51.50%)	190
18–30	969 (77.64)	279 (22.36%)	1248
31–64	2126 (77.34%)	623 (22.66%)	2749
>64	125 (57.87)	91 (42.13%)	216

Values are expressed as the mean and standard deviation (M ± SD) for continuous variables or as numbers and percentages (*n* (%)) for categorical variables. BMI, body mass index.

**Table 2:** Answers and scoring of MEDAS.

Questions	Answers	Women	Men	Whole Sample	*p*-Value
Olive oil, main dressing					0.73
	No	130 (3.92)	46 (4.22)	176 (4.00)	
	Yes	3183 (96.08)	1044 (95.78)	4227 (96.00)	
Olive oil, ≥4 ts/day					0.94
	No	1614 (48.72)	533 (48.90)	2147 (48.76)	
	Yes	1699 (51.28)	557 (51.10)	2256 (51.24)	
Vegetables, ≥2 s/day					0.0001 ***
	No	926 (27.95)	479 (43.95)	1405 (31.91)	
	Yes	2387 (72.05)	611 (65.05)	2998 (68.09)	
Fruits, ≥3 s/day					0.02 *
	No	2226 (67.19)	692 (63.49)	2918 (66.27)	
	Yes	1087 (32.81)	398 (36.51)	1485 (33.73)	
Red meat, ≤1 s/day					0.002 **
	No	1411 (42.59)	521 (47.80)	1932 (43.88)	
	Yes	1902 (57.41)	569 (52.20)	2471 (56.12)	
Butter, ≤1 s/day					0.87
	No	1522 (45.94)	497 (45.60)	2019 (45.85)	
	Yes	1791 (54.06)	593 (54.40)	2384 (54.15)	
Sweet beverage, ≤1 s/day					0.23
	No	1551 (46.82)	487 (44.68)	2038 (4629)	
	Yes	1762 (58.18)	603 (55.32)	2365 (53.71)	
Red wine, ≥7 s/week					0.0001 ***
	No	3053 (92.15)	858 (78.72)	3911 (88.82)	
	Yes	260 (7.85)	232 (21.28)	492 (11.18)	
Legumes, ≥3 s/week					0.0001 ***
	No	1735 (52.37)	468 (42.94)	2203 (50.03)	
	Yes	1578 (47.63)	622 (57.06)	2200 (49.97)	
Fish and seafood, ≥3 s/week					0.04 *
	No	2046 (51.76)	636 (58.35)	2682 (60.91)	
	Yes	1267 (38.34)	454 (41.65)	1721 (39.09)	
Sweets, ≤3 s/week					0.1
	No	1615 (48.75)	500 (45.87)	2115 (48.03)	
	Yes	1698 (51.25)	590 (54.13)	2288 (51.97)	
Nuts, ≥3 s/week					0.008 **
	No	1675 (50.56)	602 (55.23)	2277 (51.71)	
	Yes	16.38 (49.44)	488 (44.77)	2126 (48.29)	
White meat over red					0.0001 ***
	No	759 (22.91)	370 (33.95)	1129 (25.64)	
	Yes	2554 (77.09)	720 (66.05)	3274 (74.36)	
“Soffrito”, ≥2 s/week					0.0001 ***
	No	1646 (49.68)	470 (43.12)	2116 (48.06)	
	Yes	1667 (50.31)	620 (56.88)	2287 (51.94)	
Adherence to the MedDiet					
MEDAS score		7.38 ± 2.16	7.43 ± 2.23	7.39 ± 2.18	
Low		648 (19.55)	221 (20.27)	869 (19.73)	
Medium		2092 (63.15)	679 (62.30)	2771 (62.94)	
High		573 (17.30)	190 (17.43)	763 (17.33)	

The answers to the MEDAS questionnaire were compared within the same gender group (men versus men, women versus women). Data are expressed as numbers and percentages in brackets (n (%) for categorical variables. Statistical significance has been attributed to results with * *p* < 0.05, ** *p* < 0.001, and *** *p* < 0.0001. Vegetables daily serving: 1 medium portion = 200 g. Fruit daily serving: 1 serving = 100–150 g portion. Red meat/hamburgers/other meat daily serving: 1 medium portion = 100–150 g. Butter, margarine, or cream daily serving: 1 medium portion = 12 g. Sweet or sugar-sweetened carbonated beverages daily serving: 1 medium portion = 200 mL. Red wine daily serving: 1 medium portion = 125 mL. Legumes weekly serving: 1 portion = 150 g. Fish daily serving: 1 medium portion = 100–150 g. Seafood daily serving: 1 medium portion = 200 g. Nuts weekly serving: 1 portion of dairy product = 30 g. MEDAS, Mediterranean diet adherence screener; MedDiet, Mediterranean diet; s, serving; ts, tablespoon.

**Table 3:** Participants’ body composition characteristics and obesity classification at baseline.

	Men	Women	Whole Sample
N sample			
Height (cm)	175.70 ± 5.97	161.37 ± 6.31	172.20 ± 8.64
Weight (kg)	91.90 ± 15.98	68.40 ± 12.96	86.15 ± 18.31
BMI (kg/m^2^)	29.70 ± 4.60	26.30 ± 4.94	28.87 ± 4.88
Waist circumference (cm)	98.84 ± 15.99	83.24 ± 10.77	95.03 ± 16.29
Abdomen circumference (cm)	105.68 ± 12.97	94.28 ± 11.14	102.89 ± 13.42
WHR	0.94 ± 0.12	0.80 ± 0.06	0.91 ± 0.13
PhA (°)	6.26 ± 0.53	5.59 ± 0.54	6.11 ± 0.60
BCM (kg)	37.68 ± 5.22	23.80 ± 3.83	34.29 ± 7.74
LBM (kg)	59.51 ± 7.09	39.75 ± 5.02	54.67 ± 10.80
PBF (%)	28.74 ± 7.66	35.30 ± 6.94	30.34 ± 7.97
VAT (g)	632.75 ± 328.88	442.26 ± 251.84	577.29 ± 318.97
Obesity phenotype groups			
NW	21 (29.58)	6 (26.09)	27
NWO	1 (1.41)	5 (21.74)	6
MONW	2 (2.82)	0 (0)	2
MUO	23 (32.39)	8 (34.78)	31
MHO	24 (33.80)	4 (17.39)	28

Values are expressed as the mean and standard deviation (M ± SD) for continuous variables or as numbers and percentages (n (%)) for categorical variables. BMI body mass index; WHR, waist-to-height ratio; PhA, phase angle; BCM, body cell mass; LBM, lean body mass; MHO, metabolically unhealthy obese; MONW, metabolically obese normal weight; MUO, metabolically healthy obese; NOW, normal weight obese; NW, normal weight; PBF, percentage of fat mass; VAT, visceral adipose tissue.

**Table 4:** MedDiet adherence groups before and after MedDiet treatment.

	Baseline			After 8 wks of MedDiet Treatment		
	Women	Men	Whole Sample	Women	Men	Whole Sample
MEDAS score	7.29 ± 2.24	7.26 ± 2.26	7.28 ± 2.23	8.90 ± 2.21	9.30 ± 2.40	9.0 ± 2.25
Low adherence	16 (22.54)	4 (17.39)	20 (21.27)	5 (7.04)	3 (13.04)	8 (8.51)
Medium adherence	45 (63.38)	15 (65.22)	60 (63.83)	34 (47.89)	8 (34.78)	42 (44.68)
High adherence	10 (14.08)	4 (17.39)	14 (14.90)	32 (45.07)	12 (52.17)	44 (46.81)

Values are expressed as the mean and standard deviation (M ± SD) for continuous variables or as numbers and percentages (n (%)) for categorical variables. MEDAS, Mediterranean diet adherence screener; MedDiet, Mediterranean diet.

**Table 5:** Correlation matrix: correlation between variables on the whole sample.

	Δ% Weight	Δ% BMI	Δ% PhA	Δ% PBF	Δ% BCM%	Δ% MEDAS Score	Δ%MedDiet Adherence Groups
Δ% Weight	1.00	0.99	−0.06	0.10	−0.15	−0.28	−0.28
Δ% BMI	0.99	1.00	−0.06	0.10	−0.15	−0.28	−0.27
Δ% PhA	−0.06	−0.06	1.00	0.00	0.60	0.12	0.07
Δ% PBF	0.10	0.10	0.00	1.00	−0.67	−0.18	−0.19
Δ% BCM%	−0.15	−0.15	0.60	−0.67	1.00	0.24	0.20
Δ% MEDAS score	−0.28	−0.28	0.12	−0.18	0.24	1.00	0.51
Δ%MedDiet adherence groups	−0.28	−0.27	0.07	−0.19	0.20	0.51	1.00

BMI, body mass index; PhA, phase angle; PBF, percentage of fat mass; BCM, body cellular mass; MEDAS, Mediterranean diet adherence screener; MedDiet, Mediterranean diet.

**Table 6:** Correlation matrix: correlation between variables in women.

	Δ% Weight	Δ% BMI	Δ% PhA	Δ% PBF	Δ% BCM%	Δ% MEDAS Score	Δ%MedDiet Adherence Groups
Δ% Weight	1.00	1.00	−0.01	0.11	−0.13	−0.43	−0.31
Δ% BMI	1.00	1.00	−0.02	0.12	−0.15	−0.44	−0.32
Δ% PhA	−0.01	−0.02	1.00	0.08	0.55	0.11	0.06
Δ% PBF	0.11	0.12	0.08	1.00	−0.65	−0.20	−0.21
Δ% BCM%	−0.13	−0.15	0.55	−0.65	1.00	0.23	0.17
Δ% MEDAS score	−0.43	−0.44	0.11	−0.20	0.23	1.00	0.47
Δ%MedDiet adherence groups	−0.31	−0.32	0.06	−0.21	0.17	0.47	1.00

BMI, body mass index; PhA, phase angle; PBF, percentage of fat mass; BCM, body cellular mass; MEDAS, Mediterranean diet adherence screener; MedDiet, Mediterranean diet.

**Table 7:** Correlation matrix: correlation between variables in men.

	Δ% Weight	Δ% BMI	Δ% PhA	Δ% PBF	Δ% BCM%	Δ% MEDAS Score	Δ%MedDiet Adherence Groups
Δ% Weight	1.00	0.99	−0.30	0.13	−0.31	0.01	−0.08
Δ% BMI	0.99	1.00	−0.29	0.13	−0.30	0.04	−0.07
Δ% PhA	−0.30	−0.29	1.00	−0.38	0.79	0.24	0.10
Δ% PBF	0.13	0.13	−0.38	1.00	−0.80	−0.16	−0.15
Δ% BCM%	−0.31	−0.30	0.79	−0.80	1.00	0.31	0.26
Δ% MEDAS score	0.01	0.04	0.24	−0.16	0.31	1.00	0.66
Δ%MedDiet adherence groups	−0.08	−0.07	0.10	−0.15	0.26	0.66	1.00

BMI, body mass index; PhA, phase angle; PBF, percentage of fat mass; BCM, body cellular mass; MEDAS, Mediterranean diet adherence screener; MedDiet, Mediterranean diet.

## Data Availability

This published article includes all the data generated or analyzed during this study.
